# Assessment of laparoscopic instrument reprocessing in rural India: a mixed methods study

**DOI:** 10.1186/s13756-021-00976-x

**Published:** 2021-07-23

**Authors:** Daniel Robertson, Jesudian Gnanaraj, Linda Wauben, Jan Huijs, Vasanth Mark Samuel, Jenny Dankelman, Tim Horeman-Franse

**Affiliations:** 1 Department of BioMechanical Engineering, Delft University of Technology, Mekelweg 2, 2628 CD Delft, The Netherlands; 2 Shanthi Bhavan Medical Center, Biru, Jharkhand India; 3 Heart Consultancy, Renkum, The Netherlands; 4 Surgery Unit 1, Christian Medical College Hospital, Vellore, Tamil Nadu India

**Keywords:** Sterile reprocessing, Laparoscopy, LMIC, Global health

## Abstract

**Background:**

Laparoscopy is a minimally-invasive surgical procedure that uses long slender instruments that require much smaller incisions than conventional surgery. This leads to faster recovery times, fewer post-surgical wound infections and shorter hospital stays. For these reasons, laparoscopy could be particularly advantageous to patients in low to middle income countries (LMICs). Unfortunately, sterile processing departments in LMIC hospitals are faced with limited access to equipment and trained staff which poses an obstacle to safe surgical care. The reprocessing of laparoscopic devices requires specialised equipment and training. Therefore, when LMIC hospitals invest in laparoscopy, an update of the standard operating procedure in sterile processing is required. Currently, it is unclear whether LMIC hospitals, that already perform laparoscopy, have managed to introduce updated reprocessing methods that minimally invasive equipment requires. The aim of this study was to identify the laparoscopic sterile reprocessing procedures in rural India and to test the effectiveness of the sterilisation equipment.

**Methods:**

We assessed laparoscopic instrument sterilisation capacity in four rural hospitals in different states in India using a mixed-methods approach. As the main form of data collection, we developed a standardised observational checklist based on reprocessing guidelines from several sources. Steam autoclave performance was measured by monitoring the autoclave cycles in two hospitals. Finally, the findings from the checklist data was supported by an interview survey with surgeons and nurses.

**Results:**

The checklist data revealed the reprocessing methods the hospitals used in the reprocessing of laparoscopic instruments. It showed that the standard operating procedures had not been updated since the introduction of laparoscopy and the same reprocessing methods for regular surgical instruments were still applied. The interviews confirmed that staff had not received additional training and that they were unaware of the hazardous effects of reprocessing detergents and disinfectants.

**Conclusion:**

As laparoscopy is becoming more prevalent in LMICs, updated policy is needed to incorporate minimally invasive instrument reprocessing in medical practitioner and staff training programmes. While reprocessing standards improve, it is essential to develop instruments and reprocessing equipment that is more suitable for resource-constrained rural surgical environments.

**Supplementary Information:**

The online version contains supplementary material available at 10.1186/s13756-021-00976-x.

## Background

India is a low-income to middle-income country (LMIC) with a very diverse healthcare landscape. Many urban centres have world-class private facilities, but there are also public urban and rural hospitals that serve the uninsured low-income population [1]. Some of the these public centres are investing in laparoscopy to enable patients to benefit from this form of minimally invasive surgery (MIS).

Laparoscopy requires far smaller incisions than conventional surgery leading to faster recovery times, less pain, and less blood loss. Additionally, the smaller incisions used in MIS lead to a lower infection rate compared with open surgery. These advantages could greatly benefit patients in low to middle income countries as it could result in a faster return to work and a lower bed occupation in hospital wards [2]. Several studies conducted in India have compared post-surgical site infections rates of laparoscopic versus open surgery, these studies showed an infection reduction of 9.6%, 16%, and 21% in [3, 4], and [5] respectively.

However, laparoscopy is often considered too expensive and unsafe for LMIC settings [2, 6], particularly when taking into account the complex reprocessing procedures that the instruments require. Minimally invasive surgery relies on long, slender instruments containing fragile tubular components that require specialised reprocessing methods [7]. Updated training of staff and specific equipment is needed such as long brushes to remove debris from lumen and more advanced autoclaves that sterilise by ensuring steam penetration into all of the components. When instruments are not reprocessed according to manufacturer’s instructions, the advantages of laparoscopic surgery are negated by higher instrument wear and higher patient infection rates and the operating cost increases [8]. Insufficient sterilisation has led to several infection outbreaks after laparoscopic procedures [9–12].

Numerous authors have assessed the sterile processing of surgical instruments in LMIC hospitals by carrying out a checklist survey [13–15]. However, no studies exist that document the current laparoscopic reprocessing methods in LMICs. Therefore, it is unknown whether hospitals, that currently perform laparoscopic surgeries, have the updated their facilities, standard operating procedures (SOP) and training to safely reprocess laparoscopic instruments.

Therefore, the aim of this study was to assess the capacity for sterile reprocessing of laparoscopic instruments in rural India in terms of procedures, infrastructure and effectiveness of sterilisation equipment. We developed a checklist, performed measurements on the autoclaves in the hospital, and conducted interviews. By documenting the sterilisation process, we were able to determine whether the processes in these Indian rural hospitals are suitable to safely reprocess laparoscopic instruments and discover the underlying causes for these current methods.

## Methods

In March 2020, four rural hospitals in India were visited to assess the sterile reprocessing processes for laparoscopic instruments. This study used a mixed methods approach to determine the current status of the techniques used in sterile reprocessing [16]. These methods included qualitative observations and interviews and quantitative autoclave measurements. Ethical permission to perform the study was granted by the Delft University of Technology Human Research Ethics Committee (document number 1063) and written clearance was provided by each of the hospitals visited.

### Observations

The variations in reprocessing methods in the hospitals were studied by performing observations using a checklist. This developed checklist was based on (inter)national guidelines, expert recommendations and previous experiences [17–21]. An initial pilot study was run over a six day period to find missing entries in the checklist, before the final version was used for three other hospitals. After the third hospital, the checklist was updated to add missing entries and re-ordered to follow the common sterile reprocessing procedures.

The checklist was filled by conducting direct observations by one observer who visited the reprocessing departments in each hospital. The observations in the hospitals were performed during a single day, except for the pilot study. When a direct observation of an entry could not be made, the item was completed by asking the responsible person in the hospital. The final version of the checklist is added as a Additional file 1.

### Measurements

Measurements were performed on the autoclaves to determine the capabilities of the autoclaves for sterilising laparoscopic instruments. The temperature and pressure within the autoclave chamber was monitored during a sterilisation cycle, using EBRO® EBI sensors: EBI 10-T22x, EBI 10-TP230, and EBI16 [Xylem Analytics Germany Sales GmbH & Co. KG Ebro, Ingolstadt Germany]. The EBI 16 has a process challenge built-in which provides an indication of air removal during the vacuum stage. The data collected by the sensors were processed with WinlogMed® software [V3.64 2017, Xylem Analytics Germany Sales GmbH & Co. KG Ebro, Ingolstadt Germany].

### Interviews

Semi-structured interviews were conducted with surgeons and staff to gain understanding of the motives and interpretations of reprocessing methods that were used in their hospital. Three different interview guides were made for surgeons, nurses and SSD staff respectively. Surgeons were queried on the incidence of infections, nurses about their training and access to new information, and the SSD staff on failure, maintenance and repair of laparoscopic equipment. During the interviews, the subjects were presented with the methods of reprocessing the laparoscopic instruments observed in their hospital. Then they were asked to indicate the motivation for using the practiced methods, and how their reprocessing methods could be improved.

The interviews were recorded and manually transcribed and coded in ATLAS.TI [8.4.24.0, ATLAS.ti Scientific Software Development GmbH, Berlin, Germany] with one author generating the codes and coding the interviews. These codes were then used by another author who independently coded the interviews. Both authors discussed the coding of the interviews until a consensus was reached.

## Results

We received permission to assess eight hospitals, however, because of the Covid-19 pandemic, not all could be visited. We were able to conduct the checklist, in total of four hospitals. The hospitals included in the study are shown in Table 1. They were all rural, secondary district hospitals in the states: Jharkhand, Tamil Nadu and Assam.

**Table 1:** Facility size and surgical capacity

Type of hospital	Total number of beds	Operating theatres	Surgeries per year	Laparoscopic surgeries per year	Operating theatre nurses	Sterile supply department staff	Biomedical technicians
Secondary district	100	4	300	80–100	6	2	0
Secondary district	35	2	800	40–50	28	0	0
Secondary district	50	2	600	40–50	6	0	0
Secondary district	25	1	360	100–110	4	0	0

### Sterile processing capacity and facilities

Table 2 shows information on the hospitals’ infrastructure, record keeping and procedures and available equipment. Of the four hospitals evaluated, two had an area available that was designated as a Sterile Supply Department, (SSD). These were areas that are constructed such that a clear workflow could be maintained with separate areas for dirty, clean and sterile instruments. However, in neither hospital did staff follow this workflow, and only in one hospital the SSD was used as the main sterile reprocessing location.

**Table 2:** SSD information and available equipment in 4 rural hospitals

General	Hospitals n=
Record keeping of sterile processing	3
Hospitals with a SSD	2
Area with dirty to clean processing	2
instruments are processed in the SSD	1
Periodic review of sterile processing	0
Product documentation are available	0
There is a procedure for new materials/instruments	0
Disassembly instructions for instruments are available	0
There is a written protocol for manual cleaning	0
There is a protocol for repair of instruments	0
Laparoscopic instruments are processed in the SSD	0

None of the hospitals had dedicated tools and equipment for reprocessing surgical instruments. One hospital had an automated washer-disinfector, but it was not in use because not enough instruments were used during the day to fill the machine to full capacity. Also, none of the hospitals had any personal protective equipment (PPE) for staff, like waterproof gowns, thick elbow gloves or face shields. In all cases standard surgical gloves were used as the only protection.

### Laparoscopic instrument reprocessing method

**Fig. 1:**
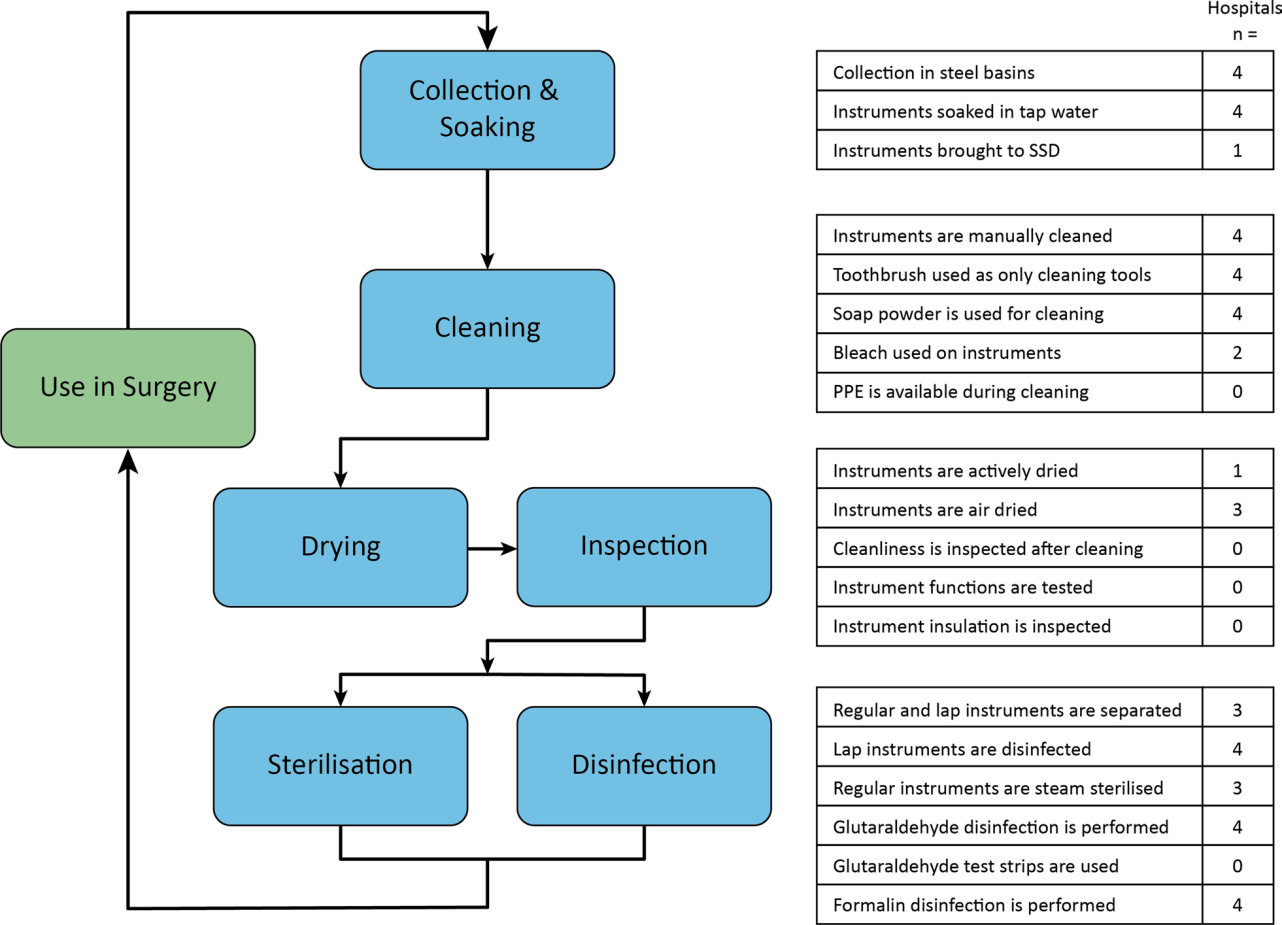
Summary of the instrument reprocessing cycles in the four hospitals. The data was collected using an observational checklist. The flow chart shows the steps in the reprocessing cycle and the table highlights details of the process

The basic steps of the reprocessing cycles along with the details observed in the hospitals are shown in Fig. 1. These steps are based on WHO and CDC sterile reprocessing manuals [17, 19]. All of the hospitals operate the same steps in the sterile reprocessing cycle, with some variations which can be seen in the details in Fig. 1.

#### Cleaning

In all four hospitals, the nurses reprocessed all the instruments during the time between surgeries. Laparoscopic and regular surgical instruments were collected together into uncovered stainless steel basins (n = 4) where they were soaked in tap water. The collected instruments were transported to a sink in rooms adjacent to the operating room (n = 3). In one hospital, the sink used to rinse the instruments was also used by the surgeons for washing hands.

All of the instruments were manually cleaned. No specific brushes for brushing long lumens on laparoscopic instruments were available, only toothbrushes and puncture devices like needles were used for cleaning. Instead, the lumens were held under running water to rinse. None of the hospitals used a dedicated detergent for instrument cleaning, instead, soap or clothes-washing powder was used. The detergent was applied where necessary and not used as a soaking agent. The instruments were soaked in bleach if the patient was known to be infected (n = 2).

#### Drying and inspecting

While preparing for sterilisation or disinfection, the laparoscopic instruments were separated from the regular instruments. In only one of the hospitals, the laparoscopic instruments were actively dried between cases by using a hairdryer. In the other facilities, the laparoscopic instruments were not dried before placing in the disinfectant and left to air dry at the end of the day.

#### Disinfecting/sterilising

Only the regular steel surgical instruments were steam sterilised, the laparoscopic instruments were high-level disinfected.

The main disinfection method for laparoscopic instruments was soaking in trays with a high level disinfectant. In all of the hospitals this was glutaraldehyde (Cidex). No hospital tested the minimal level of concentration of the disinfectant with Cidex indicator strips.

The other disinfection method used was formaldehyde gas, also known as formalin. The formalin chambers are chambers where instruments are sterilised by exposure to formaldehyde gas. The gas is released by formalin tablets that are placed in the chamber. In three hospitals, the chambers were used as a storage for cleaned instruments for surgery the following day. In one of hospitals the chambers were used as intermediate disinfection method in between surgeries. Only in one of the hospitals was the date of placement of the formalin tablets noted.

### Steam sterilisation

At the end of the day, the steel instruments that were used during the day were sterilised in the autoclave. In three out of four hospitals, there were manual autoclaves present. In one hospital, only a pressure cooker was available, which only sterilised textiles, like surgical gowns. One hospital used an ethylene-oxide (ETO) steriliser to sterilise all instruments with plastic or electric components like electrosurgical knives and disposable bipolar instruments.

**Fig. 2:**
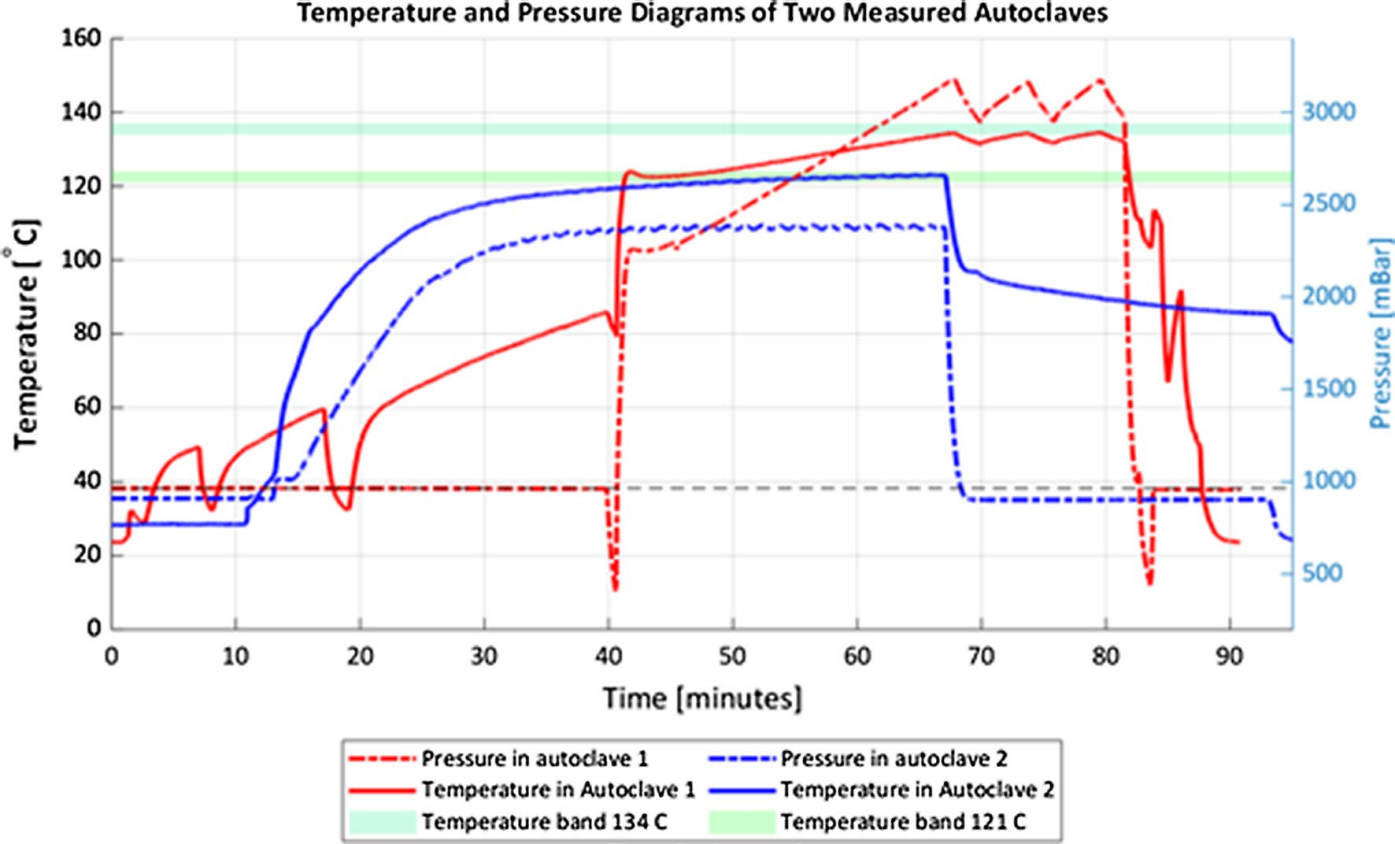
Temperature and pressure diagrams of the two measured autoclaves. The graphs show the readout of pressure and temperature sensors placed into the autoclave chamber during one cycle

In two hospitals, we performed measurements on the autoclave to assess whether the autoclave was suitable for sterilising laparoscopic equipment. Both autoclaves were manually controlled, horizontal autoclaves. The results of these measurements can be seen in Fig. 2. It shows the graphs of the pressure and lowest temperature measured in the autoclaves. Autoclave 1 sterilised at 134 C and had a very shallow vacuum phase (red dashed line), the other sterilised at 121 C and lacked a vacuum phase. Based on the readout of the sensors, the autoclaves failed to sterilise the load.

### Interview results

Interviews with 2 surgeons and 2 nurses from three different hospitals were conducted. The interview codes were categorised in three main topics, these were then further subdivided into sub-categories, this can be seen in Table 3 (Table 4).

**Table 3:** Description of available equipment in the SSDs and OT areas in the 4 rural hospitals

Description of SSD/OT cleaning facilities	Hospitals n=
Washer-disinfector	1
Drying machine	1
Water gun	0
Hand shower	0
Brushes for internal and external cleaning	0
Ultrasonic cleaner	0
Compressed air	0
Personal protection equipment	0

Clean area/sterilisation equipment	Hospitals n=
Manual steam autoclave	3
Pressure cooker	1
Ethylene oxide steriliser	1
Insulation tester	0
Microscope	0
Instrument composition baskets	0
Set Composition reference	0
Heat sealer	0

**Table 4:** Categories of interview codes

Categories	Codes in category	Frequency mentioned	Mentioned by participants
Current Methods	Explanation of Current Reprocessing methods	25	4/4
	Suggested improvements in sterilisation process	10	4/4
	Incidence of Infections and contamination	4	3/4
	Availability of written procedures for instrument cleaning	4	3/4
Financial Constraints	Availability Surgical Instruments	12	4/4
	Availability of cleaning equipment	11	4/4
Training and Education	Education of Rural Nurses	18	3/4
	Access to new information	14	4/4
	Surgeons Knowledge of Cleaning Process	12	3/4
	Education of Nurses in Developed Centres	5	2/4

#### Current methods

After presenting the observed reprocessing cycle, all respondents confirmed that the process presented by the interviewer was correct. When asked about why this process was used, two nurses confirmed that this method was taught in school and was a standard method in India.

#### Suggested improvements

Two respondents recommended more detailed training for nurses in handling the instruments.

We need to train trainers.[…] a month training program, something that helps them acquire knowledge in particular areas, [..]that nurse’s knowledge will then spread to other areas. [Nurse 2]

One surgeon did not recommend any specific improvements, but rather asked what improvements could reduce reprocessing time and costs. The other surgeon suggested the rural hospitals to have checklists for the cleaning process.

#### Infections

Three respondents (two surgeons and one nurse), indicated that they did not see any problems with the current methods because they have not had any problems with infections during laparoscopy.

We've not had what we have identified as infections […] So I think they're doing a good job. But. I need to study it to further understand and see if there are any flaws in their system. [Surgeon 1]

#### Written procedures

Both nurses said that there were no written procedures for them to follow. One surgeon mentioned that in the large urban tertiary centres, there are procedures and audits in place.

#### Availability of instruments and cleaning equipment

The financial limitation of the hospitals are reflected by the surgical instruments that are available and the means the nurses have available to clean them. The shortage of laparoscopic instruments is confirmed by all respondents and influences the procedure in different ways. Two nurses indicated that there were no replacements available when instruments break.

There is a large degree of reuse of disposable instruments and tools. Two respondents confirmed that ports, hand instruments, electrosurgical tools and even sutures were reused.

The shortage of surgical equipment also puts pressure on the time available to properly clean the instruments because the same instruments are needed in the next operation. This is confirmed by two respondents.

Speed, that's important because we need to wait between the cases so that since we only have one scope, that actually would be useful if it could to some degree be shortened. [Surgeon 2]

All respondents mentioned pieces of equipment or solutions that were financially out of reach e.g. ultrasonic cleaners, personal protection equipment, and cleaning solutions.

#### Training and education

According to a surgeon and nurses, the training given to rural nurses is a general nursing course. All respondents mention that this general nursing course is inadequate for learning how to reprocess laparoscopic instruments. The rural nurse is required to be a “*Jack of all trades”[Surgeon 1]*, they have to assist in surgery, prepare the patient, clean the operating room and reprocess the instruments. There are no specific roles given to the nurses in the hospitals where the respondents work.

#### Access to new information

Nurses also have trouble acquiring new information about new sterilisation practices and learning how to clean specific pieces of equipment. Nurses rely on the training they have received and adopt the process that is taught to them by the senior staff. If they are uncertain of something, the surgeon is the only person available to them for new information.

So if you ask a particular staff member about how to use a new handle or a new instrument, it, they don't know. […] Also, they don't know how to handle the chemical combinations they use. They are using it because they have been told. [Nurse 2]

Rural nurses would not independently change the process they already use. When asked about who should suggest changes to the sterilisation process:

It's not the surgeons task at all. He could question it, but he can't comment on it because it's taken care of by the nurses. You know, the sensible thing, if I have a problem I could always question and maybe audit it and to see if there's something going wrong with the process. A surgeon can do that. But the accountability of the process lies with the nurses. [Surgeon 1]

This surgeon also mentioned that they only get a basic level of information about reprocessing in their surgical training and that there are no courses available to update their knowledge.

## Discussion

The aim of this study was to assess whether the reprocessing facilities in rural hospitals in India were suitable to process minimally invasive surgical equipment. After evaluating the reprocessing methods in these rural hospitals, we found deficiencies in available equipment and training of staff. Although India is an ISO member, neither the ISO standards nor reprocessing procedures recommended by either WHO, CDC or other governing bodies were enforced in the rural hospitals [22].

The diversity of Indian healthcare is reflected by the presence of word class high tech hospitals as well as clinics that have to serve low income, uninsured population. Therefore, the results of this study are not representative of the whole Indian healthcare. Due to the Covid-19 pandemic, we were limited in the number of hospitals we were able to visit.

However, because of the uniformity of the results, a similar status of SSDs can be expected in many rural Indian hospitals. Similar deficiencies in training of staff and equipment have been found by several authors studying sterile reprocessing in other LMICs. [14, 15, 23] A survey of the sterile processing capacity of 59 facilities in 3 African countries by Fast et al. showed similar lack of training, PPE, detergents and reprocessing tools as found in this study [13].

Laparoscopic equipment is expensive due to the complexity of the components, therefore hospitals generally own one laparoscopic instrument set. Staff in the hospitals tried to reduce operative costs by maximising the lifespan of all pieces of equipment. Therefore, gentler reprocessing procedures, like high level disinfection, are preferred over more effective methods such as steam sterilisation. Many of the limitations of high level disinfection were not known to hospital staff.

The availability of one laparoscopic instrument set meant that the instruments had to be reprocessed in between surgeries. This severely limited the time nurses had to clean the instruments and lead to inspection and validation not being actively performed.

The nurses had 30–45 min in between cases to prepare the operating room for the next patient, and clean and sterilise the instruments. The lack of inspection after cleaning resulted in many of the instrument surfaces to still contain visual contamination. In addition, damages to components, such as the electrical insulation, might be overlooked as a result of limited inspection tools. Burns caused by insulation failure is one of the most common and severe complications during laparoscopy [24]. The lack of replacement instruments were also a cause for converting the surgery from laparoscopic to an open procedure, as indicated by one of the nurses. This increases the risk of infection.

In the peripheral hospitals, strong preconceptions exist in sterile reprocessing, because new knowledge, such as scientific literature and manufacturer’s instructions, does not reach the nurses. This impacts patient and staff safety, but is also detrimental to equipment. For instance, instruments were commonly disinfected using bleach, which has long been known to corrode surgical instruments [13].

The concentration of glutaraldehyde has to be periodically verified by using indicator strips, even within the manufacturers’ recommended expiry time of 14 days [20]. The effectivity of disinfectants is reduced by inserting wet instruments that dilute the disinfectant or by the presence of large amounts of bioburden [25]. However, none of the hospitals were familiar with this method of testing the glutaraldehyde. Glutaraldehyde also impedes cleaning as it binds proteins onto instruments which have not been sufficiently cleaned. This causes a build-up of bioburden, giving microbes a higher chance of surviving the disinfection or sterilisation process [19, 26].

The other main form of disinfection was using formaldehyde gas. This sterilant is unreliable as it is difficult to maintain the exact conditions needed for sterilisation such as the correct room humidity [27]. In the hospitals, it was impossible to maintain these conditions because of the lack of monitoring and the wide variety of containers used for formaldehyde disinfection.

Staff seemed unaware towards the risks they faced when handling soiled instruments or chemicals. The use of PPE was thought to be too cumbersome, which put staff at risk of cross contamination. Additionally, no precautions were taken to minimise contact with disinfectant chemicals. Formaldehyde and ethylene oxide are known to be carcinogenic, and glutaraldehyde has been reported to cause asthma and allergic reactions [28, 29].

### Ensuring sterile laparoscopic equipment

Laparoscopic equipment contains long narrow tubes and is considered a porous load. Hence, to successfully autoclave these long tubes, an autoclave is needed that performs vacuum air removal before injecting steam for sterilisation, according to standard EN 285 [30]. Neither of the autoclaves measured during this study was suitable for sterilising laparoscopic instruments due to a lack of deep, pulsed, vacuum cycles.

Without active air removal by steam-pulsing in deep vacuum, air remains trapped in the middle of the tube and sterilisation cannot be guaranteed. Active air removal is not only required to sterilise surgical equipment. In India, surgical gowns are reused by laundering and sterilising them in textile packs. Active air removal by means of steam-pulsing (above-atmospheric or in combination with a vacuum) is required for the steam to penetrate to the centre of a bundle of gowns, to ensure sterilisation [31, 32].

Both autoclaves measured during this study, showed a lack of adequate air removal or underpowered steam generation. Mainly because the lack of an adequate vacuum, these autoclaves are not suitable for the sterilisation of laparoscopic equipment. Textile packs require least above-atmospheric steam pulsing; performance can be yet improve with steam pulsing in combination with vacuum. There are currently many methods to validate autoclave cycles, however, most of these tests are unsuitable for rural LMIC hospitals. The existing methods are currently financially out of reach, or the tests are not critical for the manual autoclaves that are used in these hospitals. This raises the need for adequate low-cost process challenge devices for batch sterilisation monitoring in rural hospitals.

### Recommendations

Today, pre and post-operative broad spectrum antibiotics are used to reduce the risk of post-surgical wound infection. However the combination of intensive use and poor confirmation to protocols, as provided in the Instructions For Use (IFU), can lead to a high incidence of multidrug resistant bacteria such as MRSA [33], which influences surgical safety on a national level. Training programmes in sterile reprocessing for rural healthcare workers have to be compiled that take into account the wide range of responsibilities they carry. However, this will only become a priority when policy is installed at the local hospital levels up to the upper levels of government.

Naturally, the financial limitations have a severe impact on the reprocessing methods. With more financial means, hospitals can afford more of the necessary machinery, tools, and chemicals which are optimised for cleaning delicate instruments like laparoscopic instruments. However, because of the limited size of many of these hospitals, installing the internationally recommended processes and equipment will never be financially viable. Many international standards are written to ensure the highest levels in reprocessing safety for hospitals dealing with a large patient turnover. A minimum viable safety standard is needed so that it is clear up to what level processes have to be improved.

In support of this, redesign of both surgical equipment and reprocessing tools is needed such that the reliability of the reprocessing is less dependent on local knowledge and practices. Surgical instruments should be robust, repairable and easy to inspect so that the lifespan is maximised and procedures become more economical because of an increased availability of instruments [34, 35]. Reprocessing equipment is needed that can operate with a small batch of surgical instruments and that takes resource consumption, like water, into account.

## Conclusion

During this study, we investigated whether the reprocessing methods performed in rural hospitals in India were suitable for laparoscopic equipment. By using a checklist based on various established standards and recommendation, we were able to collect data that allowed us to assess the methods used in reprocessing laparoscopic equipment. Based on our observations, and measurements of the autoclaves, we can conclude that the current methods pose serious risks to patient and staff safety.

Interviews revealed that the issues facing the sterile processing of laparoscopic surgical instruments are a multi-faceted problem that cannot be easily solved with one strategy. It is evident that the lack of knowledge, training and equipment has a severe impact on how complex laparoscopic instruments are reprocessed. Since laparoscopy is becoming more widespread in many nations, we recommend that handling of complex instruments is incorporated into basic nursing training, and that specific surgical instruments and reprocessing equipment is designed that takes the local context into account.

## Supplementary Information

**Additional file 1**. Full checklist results table.

## Abbreviations

CDC: Center for Disease Control and prevention
IFU: Instructions for use
ISO: International Organisation for standardisation
LMIC: Low income to middle income country
MIS: Minimally invasive surgery
MRSA: Methicillin-resistant Staphylococcus aureus
PPE: Personal protection equipment
SOP: Standard operating procedure
SSD: Sterile supply department
WHO: World Health Organisation
