# Supplementary material for: Assessment of laparoscopic instrument reprocessing in rural India: a mixed methods study
Source: Antimicrob Resist Infect Control. 2021 Jul 23;10:109. doi: 10.1186/s13756-021-00976-x (PMC8300979; doi:10.1186/s13756-021-00976-x)
Supplement: Supplementary file 1 — Additional file 1. Full checklist results table. [file 13756_2021_976_MOESM1_ESM.pdf]

Full Checklist Results Table

General

Item	Notes	Hospitals n =
Who reprocesses the laparoscopic instruments	Nurses	4
Compliance with standards is verified against:	NABH Entry level	1
There is record keeping of sterile reprocessing	Paper	3
There is periodic review of reprocessing performance		0
Product descriptions/documentation are available for all products to be sterilized		0
There is a procedure for new materials/instruments		0
Disposables are being reprocessed and reused	Vessel sealers	4
For instruments that can be disassembled, (manufacturer) instructions are available for disassembly		0
There is a written protocol for manual cleaning		0
There is a protocol for repair of instruments		0

Cleaning facilities around the OT

Item	Notes	Hospitals n =
How often are the OT and cleaning areas cleaned?	Daily/After surgery/2x per week	2/1/1
Sinks are cleaned at the end of the day		2
**Personal protection**		
Sufficient personal protection devices are available such as		
Gloves of different materials	One type	4
Watertight aprons with long sleeves		0
Safety goggles		0
Splash screen		0
**Equipment in the OT**		
Compressed air gun		0
Water gun		0
Hand Shower		1
Ultrasonic cleaner		0
Flash autoclave		0
Compressed Air		0
Sink where instruments are rinsed		4
The disinfectant trays (for Cidex) are kept near the OT		4
What sort of disinfectant tray is used?	Stainless/plastic	2/2
How often are the disinfectant trays cleaned?	Unknown/monthly	3/1
How often are the rinsing trays cleaned?		

Description of SSD

Item	Notes	Hospitals n =
There is a CSSD		2
All surgical instruments are fully processed in the CSSD		0
The CSSD is cleaned daily		0
Sinks are cleaned at the end of the day		
There are separate rooms for dirty, clean and sterile goods		1
Transport of sterile and contaminated goods is strictly separated	Not used	0
The instruments are only passed through machine hatches		0
There is a storage area with racks	Not used	3
**Personal protection**		
Sufficient personal protection devices are available such as		
Gloves of different materials		0
Watertight aprons with long sleeves		0
Safety goggles		0
Splash screen		0
**Dirty area equipment available in CSSD**		
Water gun		0
Hand Shower		0
Sufficient materials for manual cleaning are available (brushes for external and internal cleaning)		0
Washer-disinfector	Not in use	1
Ultrasonic cleaner		0
Drying machine	Broken	1
Compressed air gun		0
**Inspection and assembly area**		
Insulation tester		0
Microscope		0
Composition reference (paper or computer)		0
There are composition basket sets of instruments		0
Instrument set compositions are documented		0
There are multiple laparoscopic instrument sets for consecutive surgeries		
Packaging materials available		
Cotton sheets		4
Crepe paper		0
Non-woven sheets		0
Sterilization paper bags		0
Sterilization pouches		1
Schimmelbush drums		4
Filter drums		0
Sterilization containers		0
Heat sealer is available for sealing pouches		1
**Sterilizing area**		

Information is available on the chemical and physical behaviour of the items to be sterilized		0
Sterilisers available		
Steam steriliser (size in liters)		3
ETO steriliser		1
Sterrad steriliser		0
Formaldehyde steriliser		0
Other		
The steam steriliser is:		
The steam steriliser has a readout		0
The steam steriliser has a vacuum cycle		2
Sterilisers with toxic gasses are given time to vent		0
**Routine sterilizer performance testing**		
How often is a Bowie Dick test carried out?	Never	
The result of the Bowie-Dick test is documented		
Routine sterilization performance testing is done by	None	
How often is maintentance performed on the autoclaves?	Yearly/When broken	2/2
Is there record keeping of the maintenance?		0
Disinfection in Cidex trays is done in the CSSD		0

Reprocessing procedure of laparoscopic equipment

Before surgery:

Item	Notes	Hospitals n =
Lap equipment is HLD'd		4
Lap equipment is sterilised		0

In surgery & pre-cleaning

Item	Notes	Hospitals n =
Gloves of OT nurses are changed for surgery after cleaning		1
After surgery, used lap instruments are stored in a soaking solution		2
What solution is this?	Tap water	4
The transport containers are leak proof, can be cleaned well	Stainless bowls	3

Transport to cleaning

Item	Notes	Hospitals n =
Laparoscopic equipment is transported to CSSD		0
Regular surgical equipment is transported to CSSD		1
Where is the lap equipment moved?	Sink outside OT	3
Equipment is kept moist	Directly cleaned/Water	2/2

Cleaning of laparoscopic equipment

Item	Notes	Hospitals n =
**Disinfecting**		
Lap equipment is disinfected before cleaning		0
**Removing gross soil**		
Whenever possible, lap instruments are disassembled.		4
Gross soil is removed by rinsing		1
Gross soil is removed using tools		4
What tools are used?	Toothbrush	4
**Manual Cleaning**		
What cleaning solution is used?	Soap powder	4
Instruments are fully disassembled		4
Cleaning solution is deep enough to submerge instruments	Running water/yes	3/1
Sufficient brushes for manual cleaning are present for external and internal cleaning)		0
The lumens are brushed (submerged)		0
The instrument tips are brushed		4
**Rinsing**		
Instruments are rinsed		4
In what basin	Sink	4
What water is used?	Tap	4
**Drying**		
Instruments are dried		1
Lumens are dried		1
Brushes are cleaned at the end of the day		0
Cleaning tools are disinfected		0

Inspection

Item	Notes	Hospitals n =
Instruments are inspected for cleanliness		0
Dirty instruments are reprocessed		2
Instruments are lubricated. Which lubricant?	Coconut oil/silicone oil	1/1
Insulation in inspected		0
Instruments are assembled		3
Instruments are being opened and spread out for functionality checks		0
Instrument set compositions are consulted during set assembly		0
Damaged instruments are removed from a set and replaced by an adequate replacement.		0

Packaging and sterilisation or high level disinfection

Item	Notes	Hospitals n =
**Steam sterilisation**		
How often are the lap instruments steam sterilised?	Never	4
**Chemical Disinfection**		
Instruments are chemically high-level disinfected		4
Gloves are changed when handling instruments in disinfectant		1
What disinfectant is used?	Cidex/Spirit	4/2
Soaking times are tracked		1
How is time tracked?		Wall clock
Number of soaks is tracked		0
When is liquid discarded?	14 days/after surg	2/2
Date of the liquid activation is noted		0
When was the liquid last discarded?	New bottle	3
Chemical indicators are present		0
Chemical indicators are used periodically		0
The lumens are filled with disinfectant		0
There is flow present in the disinfectant		0
Disinfectant is deep enough to submerge instruments		0
How are the disinfectant trays cleaned?	Wash+autoclave	1
Gloves are changed before rinsing instruments		0
Instruments are rinsed		4
What type of water is used?	Sterile saline	4
Are the lumens flushed? How?		0
How many times is the instrument rinsed	Once/Soak	3/1
Water is discarded after each use		0
**Drying**		
How are the instruments dried?	Not	4
How are the lumens dried?	Not	4
What air source is used?		
**Formalin**		
Formalin chambers are used		4
How many tablets are used		
Date of tablet placement is noted		
How long are the instruments in the chamber?		
Time in chamber is tracked		0
Chambers are opened between surgeries		1
Instruments are stored in Formalin chambers until the next day		4

Transport and storage

Item	Notes	Hospitals n =
In what state are the instruments stored at the end of the day?	Cleaned	4
Sterilised instruments are kept in storage		
